# Peptidylarginine Deiminase 4 Promotes the Renal Infiltration of Neutrophils and Exacerbates the TLR7 Agonist-Induced Lupus Mice

**DOI:** 10.3389/fimmu.2020.01095

**Published:** 2020-06-23

**Authors:** Norio Hanata, Hirofumi Shoda, Hiroaki Hatano, Yasuo Nagafuchi, Toshihiko Komai, Tomohisa Okamura, Akari Suzuki, I Ketut Gunarta, Katsuji Yoshioka, Kazuhiko Yamamoto, Keishi Fujio

**Affiliations:** ^1^Department of Allergy and Rheumatology, Graduate School of Medicine, The University of Tokyo, Tokyo, Japan; ^2^Department of Functional Genomics and Immunological Diseases, Graduate School of Medicine, The University of Tokyo, Tokyo, Japan; ^3^Laboratory for Autoimmune Diseases, RIKEN Center for Integrative Medical Sciences, Yokohama, Japan; ^4^Division of Molecular Cell Signaling, Cancer Research Institute, Kanazawa University, Kanazawa, Japan

**Keywords:** peptidylarginine deiminase 4, lupus nephritis, neutrophil, p38 mitogen-activated protein kinase, JNK-associated leucine zipper protein

## Abstract

Peptidylarginine deiminase 4 (PAD4), encoded by *PADI4*, plays critical roles in the immune system; however, its contribution to the pathogenesis of lupus nephritis remains controversial. The pathological roles of PAD4 were investigated in lupus model mice. An imiquimod (IMQ)-induced lupus model was analyzed in wild-type (WT) and *Padi4*-knockout (KO) mice. Proteinuria, serum anti-double stranded DNA (anti-dsDNA) antibody, and renal infiltrated cells were evaluated. Neutrophil migration and adhesion were assessed using adoptive transfer and adhesion assay. PAD4-regulated pathways were identified by RNA-sequencing of *Padi4* KO neutrophils. *Padi4* KO mice exhibited significant improvements in proteinuria progression compared with WT mice, whereas, serum anti-dsDNA antibody and immune complex deposition in the glomeruli showed no difference between both mice strains. *Padi4* KO mice showed decreased neutrophil infiltration in the kidneys. Adoptively transferred *Padi4* KO neutrophils showed decreased migration to the kidneys of IMQ-treated WT mice, and adhesion to ICAM-1 was impaired in *Padi*4 KO neutrophils. *Padi4* KO neutrophils exhibited reduced upregulation of p38 mitogen-activated protein kinase (MAPK) pathways. Toll-like receptor 7 (TLR7)-primed *Padi4* KO neutrophils demonstrated reduced phosphorylation of p38 MAPK and lower expression of JNK-associated leucine zipper protein (JLP), a p38 MAPK scaffold protein. Neutrophils from heterozygous *Jlp* KO mice showed impaired adhesion to ICAM-1 and decreased migration to the kidneys of IMQ-treated WT mice. These results indicated a pivotal role of PAD4-p38 MAPK pathway in renal neutrophil infiltration in TLR7 agonist-induced lupus nephritis, and the importance of neutrophil-mediated kidney inflammation. Inhibition of the PAD4-p38 MAPK pathway may help in formulating a novel therapeutic strategy against lupus nephritis.

## Introduction

Systemic lupus erythematosus (SLE) is a systemic autoimmune disease characterized by multi-organ inflammation with immune complex deposition and leukocyte infiltration. The treatment of SLE remains a huge challenge for clinicians considering the unfavorable prognosis associated with the side effects of steroids and immunosuppressive drugs. Genetic studies revealed the susceptibility of genes associated with the Toll-like receptor (TLR) pathway, interferon pathway, immune complex processing, and immune signal transduction in SLE, suggesting that the pathogenesis of SLE is based on immunological abnormalities (1). Autoantibodies are typically present many years before the diagnosis of SLE, consistent with the observation by Gower et al. (2) that deposition of immune complexes and complement precedes the inflammatory infiltrate. Therefore, leukocyte infiltration after immune complex deposition is the key process of organ damage in SLE and is thought to be a promising therapeutic target.

Peptidylarginine deiminase 4 (PAD4) is an enzyme that converts arginine residues to citrulline through post-translational modifications (3). *Peptidylarginine deiminase 4* (*PADI4*), which encodes PAD4, was initially identified as a non-HLA susceptibility gene in rheumatoid arthritis (4). *PADI4* is primarily expressed in neutrophils (5). Subsequently, extensive research revealed the contribution of *PADI4* to the pathogenesis of diverse diseases including inflammatory arthritis (3, 5), myocardial ischemia (6), and deep vein thrombosis (7). Several physiological roles associated with *PADI4*, such as pluripotency (8), p53 target gene repression (9), and regulation of the proliferation of multipotent hematopoietic cells (10) have been identified. The importance of PAD4 for innate immunity was underscored by the observation that the formation of neutrophil extracellular traps (NETs) requires PAD4 (11). In addition, citrullination regulates the functions of histones and gene transcripts. A previous study reported that PAD4 binds to transcription factors to regulate numerous gene transcriptions (12). Considering the multiple roles of PAD4 in the regulation of gene expression and immunological functions, PAD4 may be a potential target for the therapy of autoimmune diseases. However, the precise contribution of PAD4 to the pathogenesis of lupus nephritis remains unclear.

The aim of the present study was to elucidate the pathological roles of PAD4 in lupus nephritis. A TLR7 agonist, Imiquimod (IMQ)-induced SLE mouse model (13) was used to explore the pathogenesis of immune complex-mediated lupus nephritis in several previous reports (14, 15). In this model, topical treatment with IMQ cream on the ear skin induced lupus-like phenotypes, such as elevated levels of serum anti-dsDNA IgG and multi-organ injury, including glomerulonephritis with immune complex deposition (13). We studied IMQ-induced lupus model mice in *Padi4* KO background to explore the pathological roles of PAD4 in lupus nephritis.

## Materials and Methods

### Mice

*Padi4* KO mice were generated by deletion of *Padi4* exon 1 in C57BL/6 (B6) background mice (3). Heterozygous *Jlp* KO mice (*Jlp* +/– mice) were described previously (16). All mice were bred in a specific pathogen-free facility.

### IMQ-Treated Mice Experiments

IMQ cream (5%, Mochida Pharmaceutical) was administered on the skin of the left ear of age-matched 8–9-week-old female B6 WT and *Padi4* KO mice every alternate day up to 8 weeks as reported previously (13). Degree of proteinuria was evaluated semi-quantitatively using Albustix (Siemens Healthineers) every week. Serum anti-dsDNA IgG titers were measured using an anti-dsDNA antibody mouse ELISA kit (Shibayagi). Serum anti-Sm (IgG, IgA, and IgM) antibodies were detected using mouse anti-Sm Total IgG ELISA kit (Alpha Diagnostic). Matrix metalloproteinase-9 (MMP-9) concentrations in kidney supernatants were measured using a Mouse Total MMP-9 Quantikine ELISA kit (R&D Systems). Serum BUN and Creatinine was measured using QuantiChrom Urea Assay Kit (BioAssay) and Serum Creatinine Detection Kit (Arbor Assays), respectively. Renal histopathology was analyzed by hematoxylin-eosin (HE) staining and immunohistochemistry. Myeloid lineage cells in the kidneys and spleen were isolated and analyzed by flow cytometry (MoFlo XDP, Beckman Coulter).

### Histological Assessment of the Ear Skin and Kidneys

The ear skin and kidneys were excised from sacrificed IMQ-treated mice 8 weeks after the first IMQ treatment, fixed with 4% paraformaldehyde, followed by embedding in paraffin. Paraffin-embedded fragments were stained with H&E. For kidney immunohistochemistry, paraffin-embedded sections were immunostained for 1 h at 4°C with goat antiserum to mouse complement C3 primary antibody (ICN/CAPPEL), followed by staining for 1 h at room temperature with Alexa Fluor 594 Donkey anti-goat IgG secondary antibody (Invitrogen). The, slides were also immunostained with 1 h at 4°C with rabbit F (ab') 2 anti-mouse IgG primary antibody (BIO-RAD), followed by staining for 1 h at room temperature with Alexa Fluor 488 goat anti-rabbit IgG secondary antibody (Invitrogen). DAPI (Invitrogen) was used for nuclear staining.

Inflammatory cells in the ear skin were counted per high-power field. Glomerular score represents the sum of scores for glomerular inflammation, proliferation, crescent formation, and necrosis as described previously (17). Each score was graded from 0 to 4. For assessing immune complex deposition in the kidneys, fluorescence intensity was scored semiquantitatively (0: no staining, 1+: mild staining, 2+: moderate staining, 3+: high staining) and average scores were calculated as described previously (17). At least 60 glomeruli per animal were assessed by two independent investigators.

### Bone Marrow Neutrophil Isolation

Bone marrow-derived neutrophils were isolated by density gradient centrifugation. For transcriptome analysis, bone marrow neutrophils from 8-week-old female B6 WT and *Padi4* KO mice were isolated magnetically using a Neutrophil isolation kit (Miltenyi Biotec). CD11b^+^ Ly6G^+^ neutrophils were isolated at higher than 85% purity by density gradient isolation, and > 97% by magnetic isolation.

### Neutrophil Adoptive Transfer

Bone marrow-derived neutrophils extracted from age-matched female B6 WT, *Padi4* KO and *Jlp* +/– mice were stained with CellTracker Green CMFDA Dye (Thermo Fisher Scientific), and 2.5 × 10^6^ neutrophils were adoptively transferred to B6 WT control mice and B6 WT mice after IMQ treatment for 4 weeks as described previously (18). The frequencies of CellTracker Green-labeled neutrophils in the kidneys and spleen of the recipients were analyzed by flow cytometry 4 h after adoptive transfer.

### Neutrophil Adhesion Assay

Bone marrow-derived neutrophils from age-matched female B6 WT, *Padi4* KO, and *Jlp* +*/–* mice were isolated, and 3 × 10^6^/ml of neutrophils in Hank's balanced salt solution (HBSS; with Ca^2+^ and Mg^2+^) containing 20 mM HEPES and 0.1% bovine serum albumin (BSA) were incubated with or without 1 μg/ml of R848 for 1 h. In some wells, p38 MAPK inhibitor SB203580 (Sigma-Aldrich) was added 30 min prior to R848 stimulation. Furthermore, neutrophils were plated in 96-well plates coated with recombinant mouse ICAM-1/CD54 Fc chimeric protein (R&D systems) for 30 min. Supernatants was removed, and analyzed for the neutrophil adhesion to ICAM-1 using CytoTox 96 non-radioactive cytotoxicity assay (Promega) as described previously (19). Neutrophil adhesion to ICAM-1 was calculated using the ratio of neutrophils adhering to ICAM-1-coated plates to neutrophils adhering to 96-well poly-L-lysine plates (20).

### Assessment of Neutrophil Extracellular Traps

Bone marrow-derived neutrophils from age-matched female B6 WT and *Padi4* KO mice after IMQ treatment for 8 weeks were resuspended in NET medium containing RPMI 1,640, 1% BSA (Cayman Chemical), 1 mM CaCl_2_ (Cayman Chemical), 10 mM HEPES (Invitrogen), and 0.2 μM SytoxGreen (Thermo Fisher Scientific) and plated at a concentration of 3 × 10^5^/ml. Neutrophils were incubated without any stimulants overnight 37°C in 5% CO_2_. Supernatants were removed and plates were washed with PBS twice. SytoxGreen positive cells, which included NETs in the extracellular parts, were counted using a fluorescence microscope in a blinded fashion. The percentage of SytoxGreen positive cells were calculated as the average of 5 fields (×100) per well.

### Flow Cytometric Analysis

Cell staining was performed in the presence of purified rat anti-mouse CD16/32 antibody (BD). Antibodies used for staining were as follows; anti-Ly6G-PE (1A8, BD), anti-CD11b-APC (M1/70, eBioscience), anti-Ly6G-APC-Cy7 (1A8, BD), anti-Ly6C-APC-Cy7 (AL-21, BD), mouse biotin-conjugated anti-CD45 (30-F11, eBioscience), anti-streptavidin-V500 (BD), and anti-7-AAD (BioLegend).

For intracellular staining for phosphorylated p38 MAPK, 1 × 10^6^/ml of bone marrow-derived neutrophils were stimulated with 1 μg/ml of R848 (Enzo Life Sciences) for 30 min in RPMI 1640 (Invitrogen) containing 10% fetal bovine serum (FBS), 100 μg/ml of L-glutamine (Sigma-Aldrich), 100 U/ml of penicillin (Sigma-Aldrich), 100 μg/ml of streptomycin (Sigma-Aldrich), and 50 μM 2-mercaptoethanol (Sigma-Aldrich). Stimulated neutrophils were stained with anti-CD11b-APC (M1/70, eBioscience) and anti-Ly6G-APC-Cy7 (1A8, BD) antibodies in the presence of purified rat anti-mouse CD16/32 antibody (BD), fixed with Lyse/Fix Buffer (BD), and permeabilized by BD Phosflow Perm Buffer II (BD). Then, neutrophils were intracellularly stained with Alexa Fluor 488 Mouse IgG1κ isotype control (BD) or Alexa Fluor 488 mouse anti-p38 MAPK (pT180/pY182) (BD).

Flow cytometric analysis was performed using MoFlo XDP and data were analyzed using FlowJo Software (Tree star).

### Transwell Assays

Transwell assays were performed as described previously (21). Bone marrow-derived neutrophils from age-matched female B6 WT and *Padi4* KO mice were resuspended in HBSS (with Ca^2+^ and Mg^2+^) (Wako) containing 0.25% fatty acid free, low endotoxin BSA (Sigma-Aldrich), and 14 mM HEPES at a concentration of 5 × 10^6^/ml. Then, 300 μl of 1 nM recombinant mouse CXCL2 (carrier-free) (BioLegend) was loaded at the bottom of the 24-well plate (ultra-low attachment; Costar, Corning). Neutrophils in 200 μl of the above-mentioned buffer were put on the Transwell filter (polycarbonate, 3 μm pores; Millipore) and placed at the top of the plate. The plates were incubated in 37°C, in 5% CO_2_. After 1 h, the Transwell filter and medium in the lower chamber were collected. Lower chambers were filled with 300 μl of HBSS (without Ca^2+^ and Mg^2+^) (Wako) containing 5 mM EDTA and put on ice for 30 min. Additionally, 300 μl of HBSS (without Ca^2+^ and Mg^2+^) containing 5 mM EDTA was added again and the cells were collected. Cell numbers were counted and relative migration was calculated for each well.

### RNA Sequencing

Neutrophils at a concentration of 1 × 10^6^/ml were incubated in 24-well plates and left unstimulated or stimulated with 1 μg/ml of R848 for 6 h in RPMI 1,640 containing 10% FBS, 100 μg/ml of L-glutamine, 100 U/ml of penicillin, 100 μg/ml of streptomycin, and 50 μM 2-mercaptoethanol. Each sample was analyzed in duplicate. After collecting the cells and removal of the supernatant, 250 μl of PBS was added, followed by pipetting with 750 μl of TRIzol LS reagent (Life Technologies) in accordance with the manufacturer's instructions. RNA extraction was performed using an RNeasy micro kit (Qiagen) in accordance with the manufacturer's instructions. The quality of extracted RNA was confirmed using a 2,100 Bioanalyzer (Agilent) and quantified with a Qubit RNA HS assay kit (Thermo Fisher Scientific). RNA sequencing libraries were prepared using a TrueSeq RNA sample preparation kit v2 (Illumina) in accordance with the manufacturer's instructions. Paired-end sequencing was performed using a MiSeq Reagent V2 kit (Illumina) on the MiSeq system.

### Sequence Data Analysis

Raw read quality was evaluated using FastQC (https://www.bioinformatics.babraham.ac.uk/projects/fastqc/) (v0.11.7). After trimming off the adaptors using cutadapt (22) (v1.16), reads containing many low quality bases (Phred quality score <20 in >20% of the bases) or very low quality bases (Phred quality score <10) were removed using the FASTX toolkit. Mean of the total read count after QC was 1.02 × 10^6^. Filtered reads were mapped to the UCSC mm10 using STAR (v2.5.3a) (23), and the read count was calculated using HTSeq (v0.6.1p1) (24). Genes expressed in less than two samples were omitted from further analysis. We compared changes in mRNA expression before and after R848 stimulation in WT and *Padi4* KO neutrophils. Differentially expressed genes before and after R848 stimulation were analyzed using the nbinomWaldTest function in the DESeq2 (25) package (v1.16.1) for R (v3.4.0) in WT and *Padi4* KO neutrophils. Differentially expressed genes were filtered by 2-fold increase or decrease and false discovery rate <0.1, then analyzed using core analysis in Ingenuity® Pathway Analysis (IPA). We conducted comparison analysis in IPA between the results of the core analysis of WT and *Padi4* KO neutrophils. Activation Z scores were calculated using IPA comparison analysis between WT and *Padi4* KO neutrophils. Read counts were normalized as count per million and scaled by low Z-score and were plotted as a heatmap with hierarchical clustering based on Ward's Method.

PAD4-binding genes were adopted from a report that involved ChIP-chip analysis in MCF-7 (12). Gene names were converted to mouse homologs using biomaRt (26). Downregulated genes in TLR 7-primed *Padi4* KO neutrophils were defined as differentially upregulated genes (more than 2-fold increase and false discovery rate <0.1) in TLR 7-primed WT neutrophils but not in *Padi4* KO neutrophils. Overlapping genes were selected between PAD4-binding genes and downregulated genes of TLR7-primed *Padi4* KO neutrophils.

### RNA Extraction, cDNA Synthesis, and Quantitative Real-Time PCR

Total RNA from neutrophils was extracted using an RNeasy Micro kit as described in the manufacturer's instructions. RNA was reverse-transcribed to cDNA with random primers (Invitrogen) and SuperScript III (Invitrogen). To measure gene expression, quantitative real-time PCR was performed by using a CFX connect real-time PCR detection system (Bio-Rad) with a QuantiTect SYBR Green PCR kit (QIAGEN). Gene expression was normalized to β-actin expression levels as a control. Sequences of the PCR primers were as follows: *Actb* (5′- AGAGGGAAATCGTGCGTGAC-3′ and 5′- CAATAGTGATGACCTGGCCGT-3′), *Padi4* (5′-CTCTCCAGGAGTCATCGTAG-3′ and 5′-CCAACACCAGCTGATACTTT-3′), *Jlp* (5′-GAGCATGTGTTTACAGATCCACTG-3′ and 5′-CATTTTCTGAGCTTCTTCTCTCGC-3′).

### Western Blotting

Bone marrow-derived neutrophils (3 × 10^6^/ml) from age-matched female B6 WT, *Padi4* KO mice were incubated in 24-well plates and stimulated with 1 μg/ml of R848 in RPMI 1640 containing 10% FBS, 100 μg/ml of L-glutamine, 100 U/ml of penicillin, 100 μg/ml of streptomycin, and 50 μM 2-mercaptoethanol. After collecting cells and removal of the supernatant, neutrophils were homogenized in RIPA buffer (Sigma-Aldrich) containing Halt phosphatase inhibitor cocktail (Thermo Fisher Scientific) and Halt protease inhibitor (Thermo Fisher Scientific). The protein samples were heated to 95°C for 5 min in Laemmli sample buffer (Bio-Rad) containing 2-mercaptoethanol and loaded onto Mini-PROTEAN TGX precast gels (Bio-Rad). Following electrophoretic transfer of proteins to an Immobilon transfer membrane (Millipore) and membrane was blocked with PVDF blocking reagent for *Can Get Signal*® (Toyobo). The membrane was incubated overnight with primary antibodies as follows; anti-JLP (D72F4, Cell Signaling) and anti-GAPDH (D16H11, Cell Signaling). The membrane was then incubated with a secondary goat anti-rabbit IgG-HRP (Invitrogen) antibody, and specific proteins were visualized using ImageQuant LAS4010 (GE Healthcare) with ECL Select (GE Healthcare).

### Statistical Analysis

GraphPad Prism7 (GraphPad Software) was used for statistical analysis except in sequencing data. Two-tailed unpaired *t-*test was used for comparison between two groups. Kruskal–Wallis test with Dunn's multiple comparisons test for proteinuria, and one-way ANOVA with Tukey's multiple comparison test for others were used for comparison among three or more groups. P < 0.05 was considered statistically significant.

## Results

### IMQ-Induced Lupus Like Phenotypes Were Ameliorated in *Padi4* KO Mice

**Figure 1:**
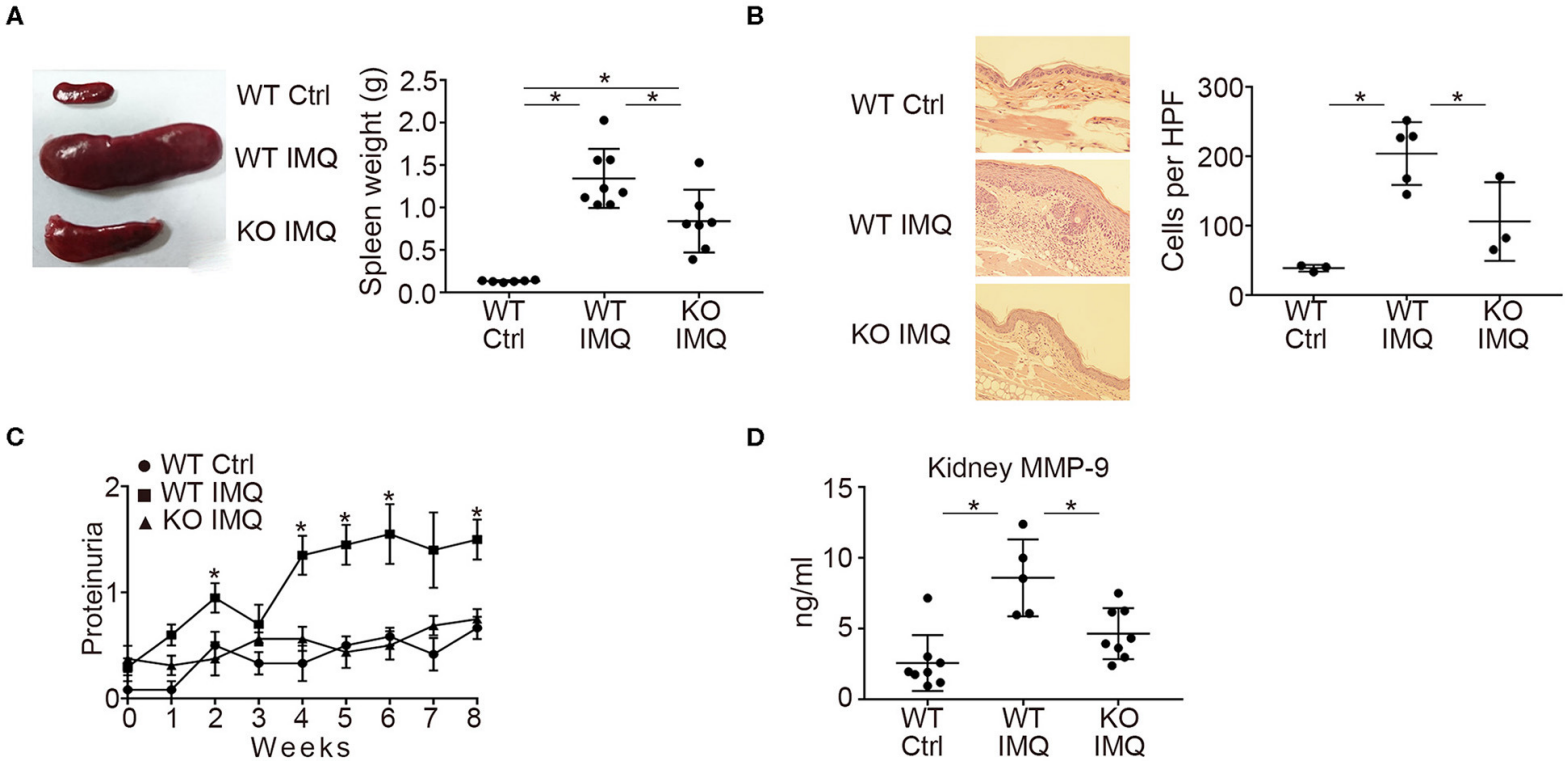
Organ involvement of an imiquimod-treated lupus model in *Padi4* KO mice. Organ involvement was analyzed in IMQ-treated WT (WT IMQ), and IMQ-treated *Padi4* KO (KO IMQ) mice 8 weeks after IMQ treatment. **(A)** Representative pictures of the spleen of WT Ctrl, WT IMQ, and *Padi4* KO IMQ mice (Left), and the spleen weights of these mice (Right) (WT Ctrl, *n* = 6; WT IMQ, *n* = 8; KO IMQ, *n* = 7). *p < 0.05 (one-way ANOVA with Tukey's multiple comparisons test). **(B)** Representative pictures of the HE staining of the ear skin (Left), and the number of inflammatory cell infiltrates in the dermis counted per high-power field from these mice (Right) (WT Ctrl, *n* = 3; WT IMQ, *n* = 5; KO IMQ, *n* = 3). *p < 0.05 (one-way ANOVA with Tukey's multiple comparisons test). **(C)** Chronological progression of proteinuria (Left) (WT Ctrl, *n* = 6; WT IMQ, *n* = 10, KO IMQ, *n* = 8). *p < 0.05 (Kruskal–Wallis test with Dunn's multiple comparisons test, *indicates a significant difference in proteinuria between WT IMQ and KO IMQ mice). **(D)** MMP-9 concentrations in kidney supernatants (WT Ctrl, *n* = 8; WT IMQ, *n* = 5; KO IMQ, *n* = 8). *p < 0.05 (one-way ANOVA with Tukey's multiple comparisons test). Data are representative of at least three independent experiments. All error bars represent ± SD except for proteinuria progression (±SEM).

First, we compared the lupus-like phenotypes induced by topical treatment of IMQ between WT and *Padi4* KO mice. The spleen weight was almost equivalent between untreated WT and *Padi4* KO mice (Figure S1). IMQ-treated *Padi4* KO (*Padi4* KO-IMQ) mice showed a significant reduction in splenomegaly compared with IMQ-treated WT (WT-IMQ) mice (Figure 1A). Epidermal hyperplasia and dermal immune cell infiltration in the ear skin of WT-IMQ mice were minimized, and cellular infiltrations were rarely observed in *Padi4* KO-IMQ mice (Figure 1B). Moreover, proteinuria was significantly ameliorated in *Padi4* KO-IMQ mice compared with WT-IMQ mice (Figure 1C). The serum BUN and creatinine showed no difference between IMQ-treated WT and *Padi4* KO mice (Figure S2). The concentrations of MMP-9, a candidate biomarker of renal damage (27), in kidney extracts of *Padi4* KO-IMQ mice were significantly decreased compared with WT-IMQ mice (Figure 1D). Spontaneous NETosis was observed in the bone marrow neutrophils of WT-IMQ mice, and was totally abrogated in *Padi4* KO-IMQ mice (Figure S3). Taken together, in *Padi4* KO-IMQ mice, lupus-like organ damage was ameliorated compared with WT-IMQ mice.

### Neutrophil Infiltration in the Kidneys Was Decreased in *Padi4* KO-IMQ Mice

**Figure 2:**
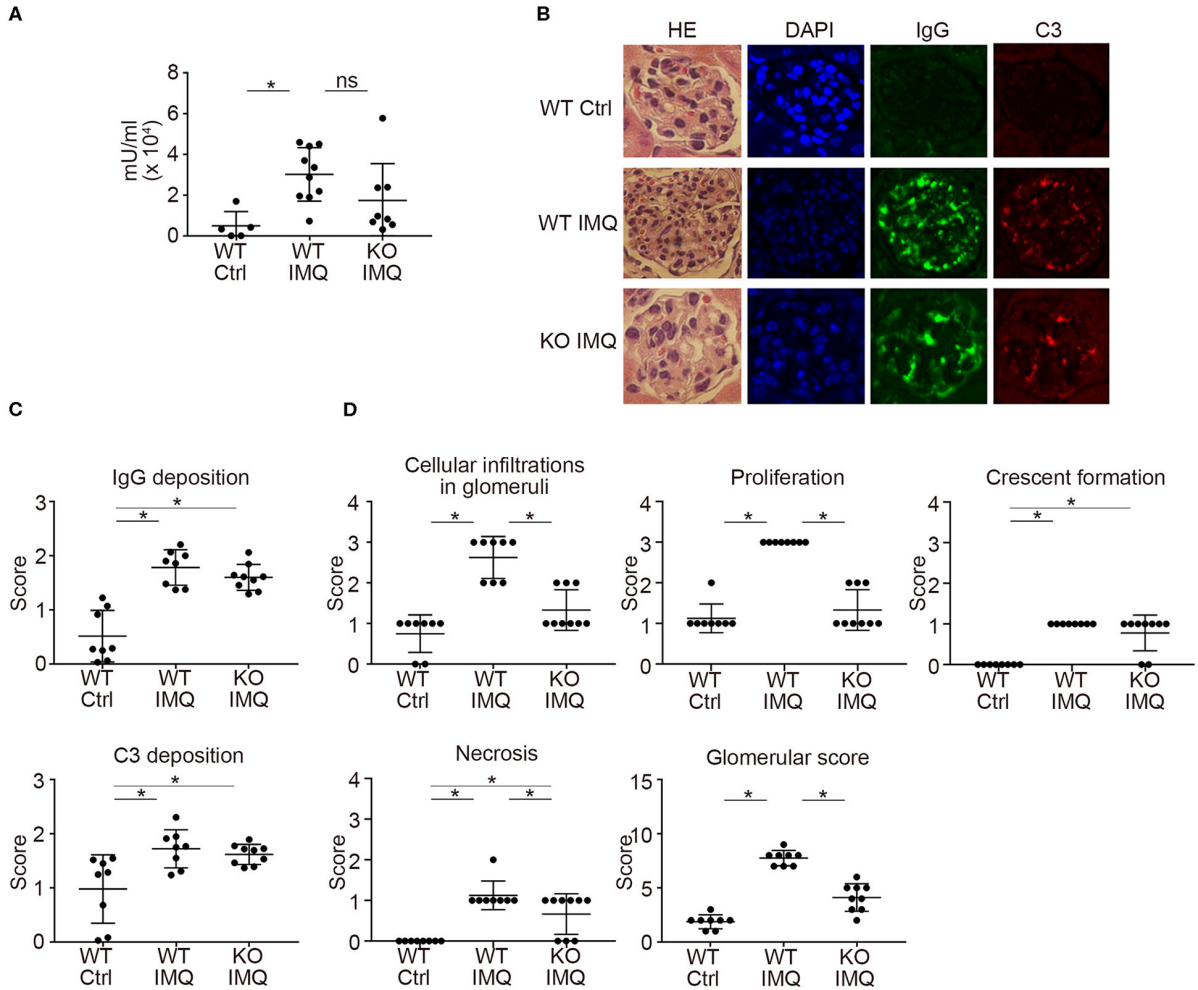
Histological analysis of the glomeruli in IMQ-treated *Padi4* KO mice. **(A)** Serum titers of anti-dsDNA antibody in the serum of WT Ctrl, WT IMQ, and KO IMQ mice for 4 weeks are indicated (WT Ctrl, *n* = 5; WT IMQ, *n* = 10; KO IMQ, *n* = 8). *p < 0.05 (one-way ANOVA with Tukey's multiple comparisons test); ns, non-significant. **(B–D)** The glomeruli of the kidneys of WT Ctrl, WT IMQ, and KO IMQ mice were analyzed histologically 8 weeks after IMQ treatment. **(B)** Representative pictures of HE, DAPI, IgG, and C3 staining. **(C)** Scores of IgG deposition and C3 deposition in the kidneys. Fluorescence intensity was scored semiquantitatively (0: no staining, 1+: mild staining, 2+: moderate staining, 3+: high staining) and average scores of at least 60 glomeruli per animal were calculated. *p < 0.05 (one-way ANOVA with Tukey's multiple comparisons test). **(D)** Glomerular score consisting of glomerular inflammation (cellular infiltration in glomeruli), proliferation, crescent formation, and necrosis of the kidneys (WT Ctrl, *n* = 8; WT IMQ, *n* = 8; KO IMQ, *n* = 9). *p < 0.05 (one-way ANOVA with Tukey's multiple comparisons test). All error bars represent ± SD.

Pertaining to the immunological characteristics, there was no difference in serum anti-dsDNA IgG and anti-Sm titers between WT-IMQ and *Padi4* KO-IMQ mice (Figures 2A, S4). Immunohistochemical analysis of the kidneys revealed that the degree of immune complex depositions were identical in WT-IMQ and *Padi4* KO-IMQ mice (Figures 2B,C). In contrast, *Padi4* KO-IMQ mice exhibited reduced glomerular scores, especially in view of cellular infiltration, compared with WT-IMQ mice (Figures 2B,D). In both WT-IMQ and *Padi4* KO-IMQ mice, cellular infiltration into the glomeruli was predominant, and infiltration into the tubulointerstitium was hardly observed (data not shown).

**Figure 3:**
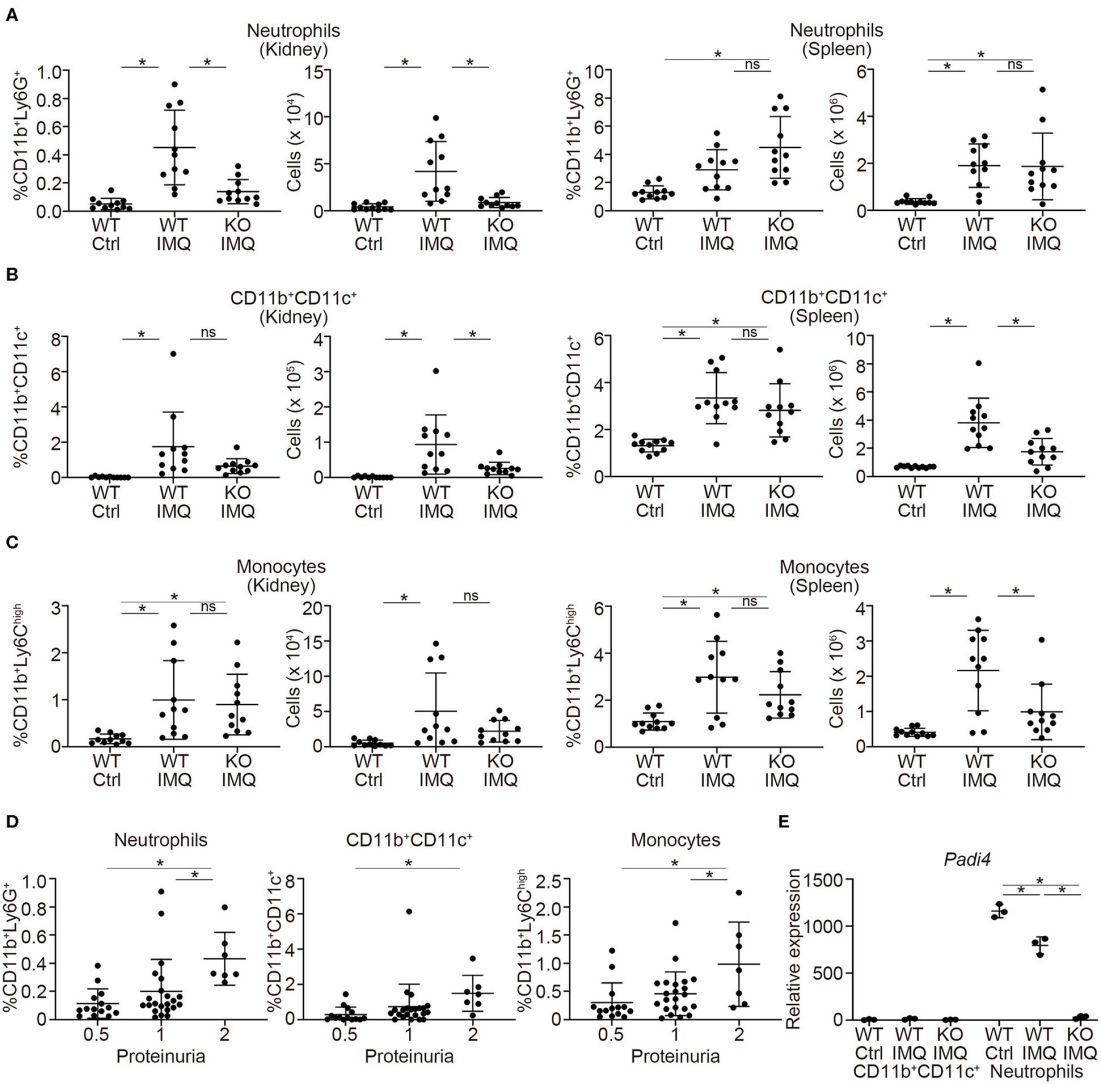
Myeloid cell infiltration in the kidneys of IMQ-treated *Padi4* KO mice. **(A–C)** The frequencies (Left) and the numbers (Right) of CD11b^+^Ly6G^+^ cells (neutrophils) **(A)**, CD11b^+^CD11c^+^ cells **(B)**, and CD11b^+^Ly6^high^ cells (monocytes). **(C)** in the kidneys and spleen 7AAD^−^ cells 8 week after IMQ treatment (WT Ctrl, *n* = 11; WT IMQ, *n* = 11; KO IMQ, *n* = 11). Data were analyzed by flow cytometry. *p < 0.05 (one-way ANOVA with Tukey's multiple comparisons test); ns, non-significant. **(D)** The percentage of myeloid cells infiltrating the kidney in each semi-quantitatively measured proteinuria (0.5, 1, and 2) (WT Ctrl, *n* = 14; WT IMQ, *n* = 14; KO IMQ, *n* = 15). *p < 0.05 (one-way ANOVA with Tukey's multiple comparisons test). **(E)** Real-time PCR analysis of the expression of *Padi4* mRNA in CD11b^+^CD11c^+^ cells (Left), and neutrophils (Right) in the kidneys (*n* = 3 per group). Results were normalized to *Actb*. *p < 0.05 (one-way ANOVA with Tukey's multiple comparisons test). Data are representative of at least three independent experiments. All error bars represent ± SD.

Flow cytometry analysis revealed a significant reduction in the numbers of myeloid cells, such as neutrophils (CD11b^+^Ly6G^+^) and CD11b^+^CD11c^+^ cells in the kidneys of *Padi4* KO-IMQ mice (Figures 3A,B). Although the frequencies of these myeloid cells in the spleens were similar between WT-IMQ and *Padi4* KO-IMQ mice, the numbers of CD11b^+^CD11c^+^ cells and monocytes (CD11b^+^Ly6C^high^) decreased in the spleen of *Padi4* KO-IMQ mice (Figures 3A–C), which may partially explain the improvement of splenomegaly in *Padi4* KO-IMQ mice shown in Figure 1A. The frequencies of CD11b^+^CD11c^+^ cells and monocyte were similar between untreated WT and *Padi4* KO mice (Figure S5). Even though the frequency of neutrophils was increased in *Padi*4 KO mice (Figure S5), the range of the frequencies was much lower. The frequencies of neutrophils and monocytes infiltrating the kidney were higher in the group of the high levels of proteinuria (Figure 3D). Considering neutrophils were decreased in the kidney of the *Padi4* KO-IMQ mice in Figure 3A, neutrophils were thought to play pivotal roles in the pathogenesis of lupus nephritis. Kidney neutrophils highly expressed *Padi4* mRNA, whereas CD11b^+^CD11c^+^ cells showed limited *Padi4* expression (Figure 3E). Interestingly, *Padi4* mRNA expression was significantly decreased in the kidney neutrophils of WT-IMQ mice compared with those of WT-control mice (Figure 3E). Taken together, the significant decrease in renal neutrophil infiltration was closely associated with the amelioration of proteinuria in *Padi4* KO-IMQ mice.

### *Padi4* KO Neutrophils Reduced the Migration Capacity to the Kidneys

**Figure 4:**
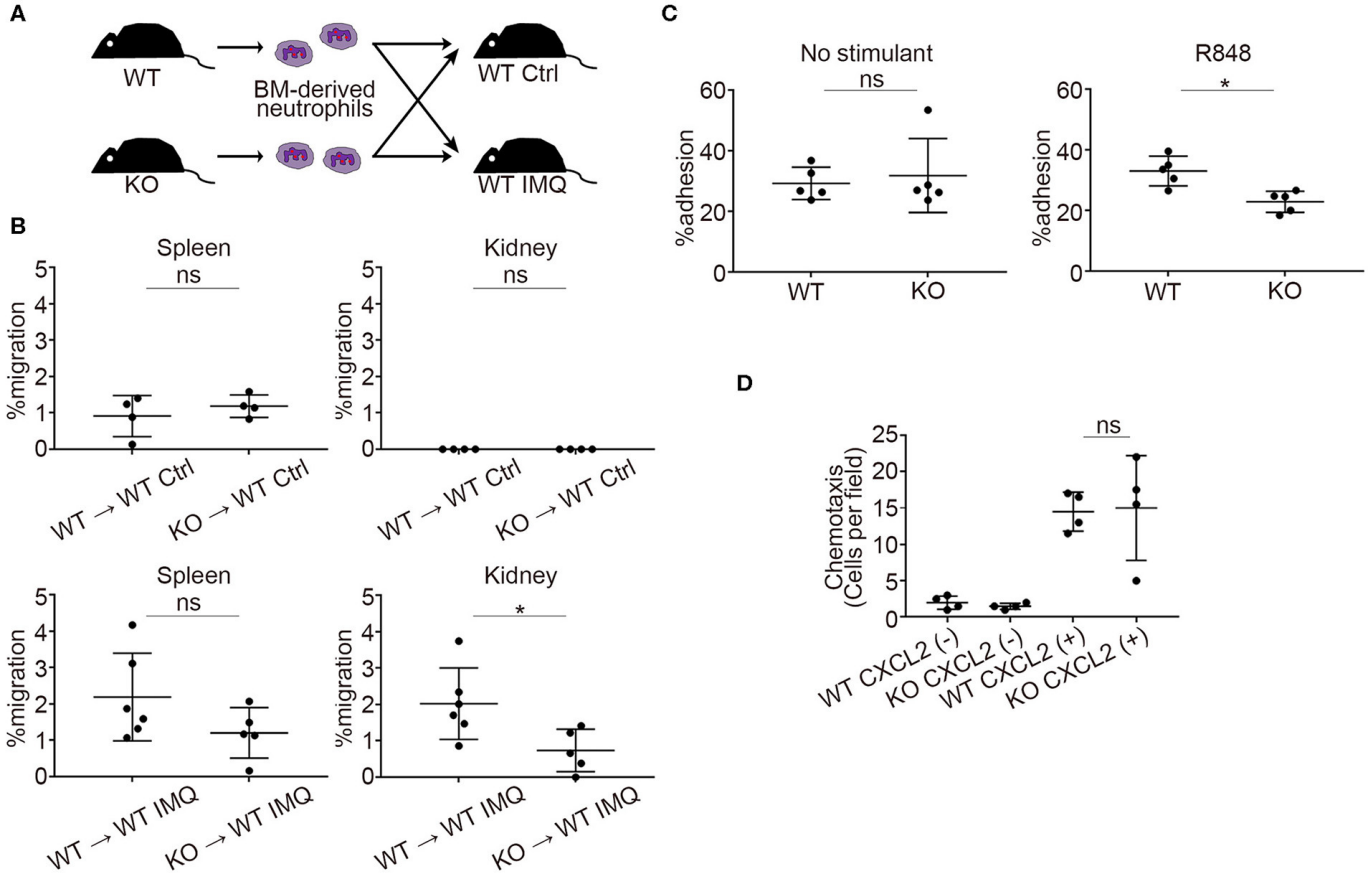
*Padi4* KO neutrophil migratory capacity *in vitro* and *in vivo*. **(A)** A schematic explanation of the adoptive transfer experiments. Bone marrow-derived neutrophils (2.5 × 10^6^) from WT and *Padi4* KO mice stained with Cell Tracker Green were adoptively transferred to WT Ctrl and WT IMQ mice 4 weeks after IMQ treatment. **(B)** The frequencies of migrated Cell Tracker Green-positive neutrophils of 7AAD^−^CD45^+^CD11b^+^Ly6G^+^ in the spleen (Left) and the kidneys (Right) (WT Ctrl for WT neutrophils and KO neutrophils, *n* = 4 for each; WT IMQ, *n* = 6 for WT neutrophils and *n* = 5 for KO neutrophils). *p < 0.05 (two-tailed unpaired *t-*test); ns, non-significant. **(C)** Adhesion of bone marrow-derived WT and *Padi4* KO neutrophils (3 × 10^6^/ml) to plates coated in ICAM-1 molecules with 1 μg/ml of R848. Cell adhesion was evaluated using the ratio of cells adhered to 96-well poly-L-lysine plates (*n* = 5 for each group). *p < 0.05 (two-tailed unpaired *t-*test); ns, non-significant. **(D)** Chemotaxis of bone marrow-derived neutrophils (1 × 10^6^) from WT and *Padi4* KO mice was assessed to CXCL2 (1 nM) (*n* = 4 per group) (one-way ANOVA with Tukey's multiple comparisons test, only the result of the analysis between WT CXCL2(+) and KO CXCL2(+) is shown.); ns, non-significant. Data are representative of at least three independent experiments. All error bars represent ± SD.

Next, we investigated the role of *Padi4* in neutrophil migration to the kidneys using adoptive transfer experiments (Figure 4A). There was a significant decrease in *Padi4* KO neutrophil migration to the kidneys compared with WT neutrophils, whereas there was no difference between WT and *Padi4* KO neutrophil migration to the spleen (Figure 4B). ICAM-1 is a representative adhesive molecule in neutrophil recruitment to the kidneys and is associated with lupus nephritis (28, 29). *Padi4* KO neutrophils showed decreased adhesion to ICAM-1 after TLR7 simulation (Figure 4C). CXCL2 is a representative renal neutrophil-attracting chemokine (30). *Cxcl2* mRNA was expressed equivalently in the kidneys of WT-IMQ and *Padi4* KO-IMQ mice (data not shown). In Transwell assays, there were no differences in chemotaxis between WT and *Padi4* KO neutrophils toward CXCL2 *in vitro* (Figure 4D). Therefore, neutrophil adhesion and migration capacity to the kidneys were regulated by *Padi4*, consistent with the decreased renal neutrophil infiltration in *Padi4* KO-IMQ mice.

### *Padi4* Promoted the Neutrophil p38 MAPK Pathway After TLR7 Stimulation

**Figure 5:**
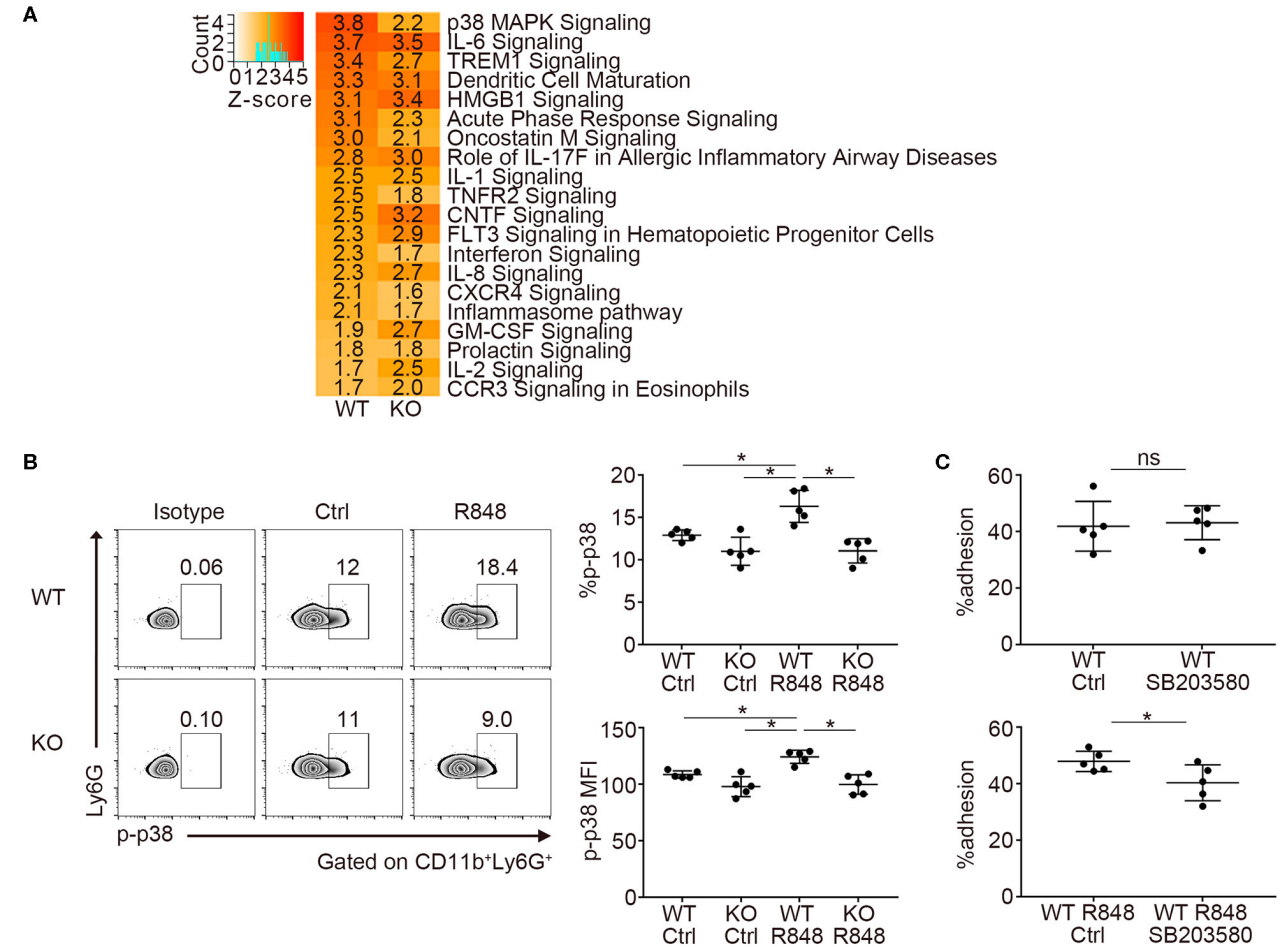
Transcriptome analysis of *Padi4* KO neutrophils. **(A)** Bone marrow-derived neutrophils were incubated with or without 1 μg/ml of R848 for 6 h. We detected differentially expressed genes before and after R848 stimulation in WT and *Padi4* KO neutrophils, respectively. The activation Z-scores in the canonical pathway analysis were calculated using IPA software. The top 20 pathways are listed. Activation Z-scores are displayed in each column. **(B)** Bone marrow-derived neutrophils (3 × 10^6^/ml) were stimulated with 1 μg/ml of R848 for 30 min and were intracellularly stained with isotype control or anti-phosphorylated p38 MAPK (p-p38 MAPK) antibodies and analyzed using a flow cytometer (*n* = 5 for each group). *p < 0.05 (one-way ANOVA with Tukey's multiple comparisons test). **(C)** R848-stimulated bone marrow-derived WT neutrophils (3 × 10^6^/ml) were incubated on ICAM-1-coated plates with or without SB203580 prior to R848 stimulation. Neutrophil adhesion to ICAM-1 was calculated using the ratio of cells adhered to 96-well poly-L-lysine plates (*n* = 5 for each group). *p < 0.05 (two-tailed unpaired *t*-test). Data are representative of at least three independent experiments. All error bars represent ± SD.

Next, we investigated the transcriptome of TLR7-stimulated neutrophils by RNA-sequencing. We focused on the changes in mRNA expression before and after R848 stimulation in WT and *Padi4* KO neutrophils, because there was a decrease in the adhesion of *Padi4* KO neutrophils to ICAM-1 after TLR7 stimulation. *Padi4* KO neutrophils showed impaired upregulation of several signaling pathways after TLR7 stimulation (Figure 5A). In particular, p38 mitogen-activated protein kinase (MAPK) signaling pathway exhibited a clear reduction in *Padi4* KO neutrophils (Figures 5A, S6). The p38 MAPK pathway regulates various neutrophil functions including adhesion (31). Indeed, the phosphorylation of p38 MAPK was significantly decreased in *Padi4* KO neutrophils compared with WT neutrophils following TLR7 stimulation (Figure 5B). Consistent with this, the α and β isoforms of the p38 MAPK inhibitor SB203580 inhibited the adhesion of WT neutrophils to ICAM-1 following TLR7 stimulation (Figure 5C). To summarize, *Padi4* promoted upregulation of the p38 MAPK pathway after TLR7 stimulation, and the p38 MAPK pathway contributed to neutrophil adhesion after TLR7 stimulation.

### *Padi4* Regulated the Expression of JLP, a p38 MAPK Scaffold Protein

**Figure 6:**
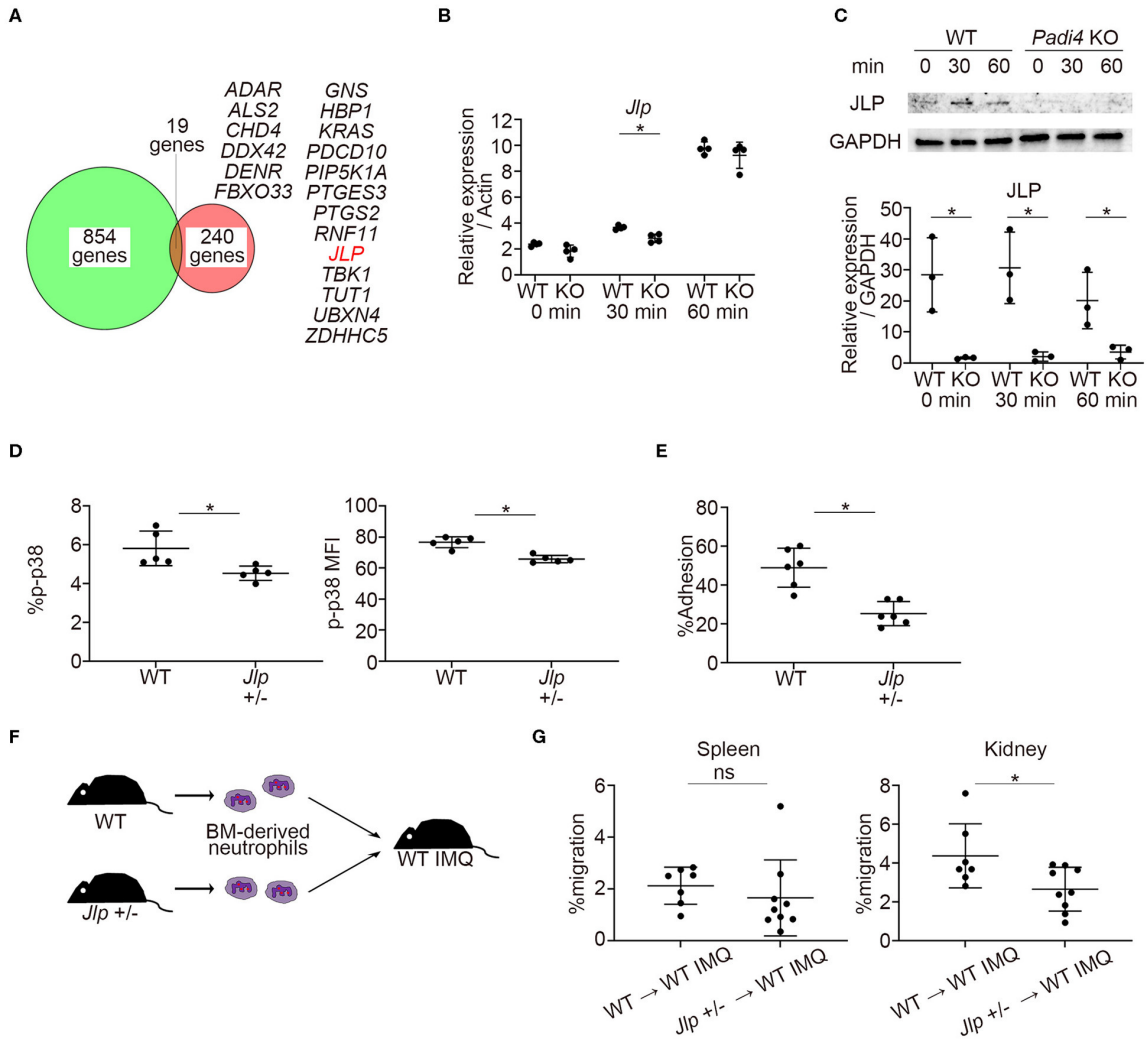
Identification of PAD4-dependent gene, *Jlp*, which encodes JLP, a scaffold protein of p38 MAPK. **(A)** Venn diagram of PAD4 Chip-chip binding genes and downregulated genes of TLR7-primed *Padi4* KO neutrophils. Downregulated genes of TLR7-primed *Padi4* KO neutrophils were defined as differentially upregulated genes (more than 2-fold increase and false discovery rate <0.1) in TLR7-primed WT neutrophils but not in *Padi4* KO neutrophils. Nineteen overlapping genes are listed in alphabetical order. **(B)** Real-time PCR analysis of the expression of *Jlp* mRNA in WT and *Padi4* KO neutrophils at 0, 30, and 60 min after R848 stimulation (*n* = 4 per group). Results were normalized to *Actb*. *p < 0.05 (two-tailed unpaired *t*-test) **(C)** Western blotting of JLP and GAPDH in WT and *Padi4* KO neutrophils at 0, 30, and 60 min after R848 stimulation. Relative expression of JLP protein (normalized to GAPDH) was analyzed between WT and *Padi4* KO neutrophils (*n* = 3 per group). *p < 0.05 (two-tailed unpaired *t*-test) **(D)** The bone marrow-derived neutrophils (3 × 10^6^/ml) of WT and *Jlp* +/– mice were intracellularly stained with isotype control or anti-phosphorylated p38 MAPK (p-p38 MAPK) antibodies and analyzed using flow cytometry (*n* = 5 for each group). *p < 0.05 (one-way ANOVA with Tukey's multiple comparisons test). **(E)** The adhesion of bone marrow-derived WT and *Jlp* +/– neutrophils (3 × 10^6^/ml) to plate-bound ICAM-1 molecules. Cell adhesion was evaluated using the ratio of cells adhered to 96-well poly-L-lysine plates (*n* = 5 for each group). *p < 0.05 (two-tailed unpaired *t-*test). **(F)** A schematic explanation of the adoptive transfer experiments. Bone marrow-derived neutrophils (2.5 × 10^6^) from WT and *Jlp* +/– mice stained with Cell Tracker Green were adoptively transferred to WT IMQ mice 4 weeks after IMQ treatment. **(G)** The frequencies of migrated Cell Tracker Green-positive neutrophils of 7AAD^−^CD45^+^CD11b^+^Ly6G^+^ in the spleen (Left) and the kidneys (Right) (*n* = 7 for WT neutrophils and *n* = 9 for *Jlp* +/– neutrophils). *p < 0.05 (two-tailed unpaired *t*-test); ns, non-significant. Data are representative of at least three independent experiments. All error bars represent ±SD.

Previous reports demonstrated that PAD4 binds to the histone and transcriptional factor complex at the promoter regions and works as a transcriptional coactivator (12). We examined the overlap between PAD4-binding genes detected by chromatin immunoprecipitation coupled with a promoter tiling array (ChIP-chip) analysis reported in MCF-7 cells (12) and downregulated genes of TLR7-primed *Padi4* KO neutrophils. Nineteen overlapping genes were identified, and the *Jlp* gene, encoding JNK-associated leucine zipper protein (JLP), was included among these (Figure 6A). JLP is a key scaffold protein for the activation of the p38 MAPK pathway (32). Indeed, mRNA expression of *Jlp* and protein expression of JLP were reduced in *Padi4* KO neutrophils after TLR7 stimulation (Figures 6B,C). mRNA expression of *Jlp* was also suppressed following LPS stimulation (Figure S7). In *Jlp* +/– neutrophils (Figure S8), phosphorylation of p38 MAPK (Figure 6D) and neutrophil adhesion to ICAM-1 *in vitro* (Figure 6E) were significantly decreased. To analyze the *in vivo* migratory capacity of the *Jlp* +/– neutrophils to the kidneys, WT and *Jlp* +/– neutrophils were adoptively transferred to WT-IMQ mice (Figure 6F). There was a significant decrease in *Jlp* +/– neutrophil migration to the kidneys compared with WT neutrophils, whereas there was no difference between WT and the *Jlp* +/– neutrophil migration to the spleen (Figure 6G). Taken together, *Padi4* promoted JLP expression after TLR7 stimulation, which led to activation of the p38 MAPK pathway and neutrophil adhesion to ICAM-1.

## Discussion

Our study demonstrated the pivotal role of PAD4 in neutrophil infiltration to the kidneys with immune complex deposition. In lupus nephritis, immune complex deposition triggers several immunological reactions, such as cellular infiltration and the activation of immune cells, including neutrophils (33). Neutrophils stimulated with immune complexes injure renal tissue via the production of reactive oxygen species, cytokines, and chemokines along with degranulation and the release of MMPs and elastase (33). Our study demonstrated that neutrophil infiltration to the kidneys was significantly suppressed in *Padi4* KO IMQ-mice, even though immune complexes were equivalently deposited. Consistent with our observations, lupus-prone MRL-*Fas*^*lpr*/*lpr*^ (MRL/*lpr*) mice lacking ICAM-1 or lymphocyte function-associated antigen-1 showed amelioration of glomerulonephritis, which was correlated with a decrease in neutrophil accumulation in the glomeruli (34, 35). In humans, neutrophils are one of the major infiltrated cells in the kidneys in lupus nephritis (36). In addition, the blood transcriptome profiles of adult lupus patients revealed an association between neutrophil signatures and lupus nephritis (37). Our observations provide an experimental evidence for the linkage between neutrophil and lupus nephritis. In our data, concentrations of MMP-9, a candidate biomarker of renal damage (27), in kidney extracts of *Padi4* KO-IMQ mice were significantly decreased compared with WT-IMQ mice. In a rat model of membranous nephropathy, a correlation between increased expression of MMP-9 within the visceral epithelial cells and the proteinuria was reported (38). Also in NZBxNZW F1 lupus mice, MMP-9 contributed to increase glomerular proteolytic activity around the onset of proteinuria (39). Additionally, glomerular MMP-9 staining were increased in the mesangial region and its levels correlated the glomerular cell proliferation in human lupus nephritis (40). Thus, MMP-9 is associated with renal impairment, especially with glomerular inflammation and proliferation. As neutrophils are known as a source of MMP-9 (41), the result might be due to a decrease in infiltration of neutrophils into the kidney of *Padi4* KO-IMQ mice. In fact, neutrophil extracellular trap-bound MMP-9 induces endothelial cells death and vascular dysfunction (42). Therefore, we selected MMP-9 as renal damage marker representing the degree of proteinuria and the renal infiltrations of myeloid cells that are characteristic of this mice model of lupus. Further investigation is needed to determine whether MMP-9 secreted from neutrophils directly contributes to renal damage in this mouse model.

The roles of PAD4 in neutrophils have been investigated. A previous study showed that there was no difference in neutrophil functions, such as phagocytosis capability, generation of reactive oxygen species, or in absolute neutrophil counts between WT and *Padi4* KO mice (43). Some recent reports suggested an association between NETosis and lupus nephritis (44, 45). NETs in SLE patients contain autoantigens, such as self-DNA, and form immune complexes to activate plasmacytoid dendritic cells (46). Because PAD4 is required for NETosis (11), the amelioration of lupus-like phenotypes in *Padi4* KO mice can be partially explained by the lack of NETosis. Nevertheless, the pathological roles of NETosis in this IMQ-induced model of lupus remains unclear. Apart from NETosis, our study demonstrated that adhesion was impaired in *Padi4* KO neutrophils, especially after TLR7 stimulation. In addition, *Padi4* KO neutrophils reduced the expression of JLP and impaired upregulation of the p38 MAPK pathway after TLR7 stimulation. Several reports suggested an alteration of integrin affinity via p38 MAPK signaling. Mice with aged neutrophils had altered integrin affinity in p38 MAPK-dependent pathways (47). In human neutrophils, p38α specifically contributes to LPS-induced adhesion (31). Moreover, p38 MAPK inhibitors decreased proteinuria in MRL/*lpr* mice (48). In this way, we supposed that the PAD4-p38 MAPK pathway and regulation of neutrophil adhesion may be promising therapeutic targets against lupus nephritis. Further study is needed to elucidate the connection between p38 MAPK pathway in neutrophils and the defect in adhesion to ICAM-1, including the possibility that expression of activated LFA-1 or Mac-1 is diminished on the surface of *Padi4* KO neutrophils. Also, further *in vivo* study is needed using conditional knockout mice in which p38α expression is specifically ablated in neutrophils. *Padi4* mRNA expression was significantly decreased in the kidney neutrophils of WT-IMQ mice compared with those of WT-control mice. Some negative feedback system may exist in the regulation of *Padi4* expression, however, additional investigation is needed to unveil its detailed mechanism. Among the identified nineteen genes, the other genes could be related to the activation of MAPK pathway. According to the literature review, four candidate genes were listed; *Kras, Rnf11, Hbp1*, and *Ptgs2*. *Kras* encodes RAS, a guanosine triphosphate (GTP)-binding protein. When stimulated by binding GTP, various signaling pathways are activated (49). Of interest, KRAS mutation was reported in SLE patient (50). *Rnf11* encodes RING finger protein 11 (RNF11), involved in various pathways, such as regulation of signal transduction, trafficking and modulation of the transcriptional activity (51). miR-200a-5p and its target gene *Rnf11* is involved in selenium deficiency-induced cardiac necrosis via MAPK activation (52). *Hbp1* encodes HMG-box transcription factor 1 (HBP1), which is involved in transcriptional repressor and cell cycle inhibitor (53). HBP1 is downstream target in p38 MAPK pathway (53). *Ptgs2* encodes cyclooxygenase 2 (COX2), and COX2 is also downstream target in p38 MAPK pathway (54). We selected *Jlp* for the further study because *Jlp* extensively regulated p38 MAPK-related genes compared to other candidate genes. Additional investigation is required to determine whether the other candidate genes control MAPK pathway in the PAD4-dependent manner.

The effectiveness of PAD4 inhibition for lupus models remains controversial. These conflicting data were due to the usage of a pan-PAD inhibitor, which also inhibits all PADs. Another important reason was the difference in the models of lupus mice and mice strains. A previous study showed that *Padi4-*deficiency and the pan-PAD inhibitor Cl-amidine (55) did not ameliorate nephritis in MRL/*lpr* mice and another lupus model induced by the transfer of SLE sera to FcγRIIAγ^−/−^Mac1^−/−^ mice, respectively (56). Additionally, Kienhöfer et al. (57) reported nephritis aggravated in a pristane model in the absence of PAD4. In contrast, Cl-amidine exhibits clear therapeutic efficacy in two lupus-prone strains, MRL/*lpr* mice (44) and New Zealand Mixed 2328 mice (45). Particularly, a recent study demonstrated that *Padi4* deficiency ameliorated immunological and clinical phenotypes of IMQ-treated mice in the FVB background (58). They attributed less kidney injury to reduced NETosis and associated vascular damage. They also observed an abrogated T cell response and a decrease of autoantibodies in *Padi4* KO mice, which was different form our observation. However, IMQ induces strain dependent responses, at least in dermatitis, and CD4^+^ T cell-associated gene expression was weaker in the B6 background (59). Therefore, we speculated that the controversial results depended on the different characteristics of the mice strains. We thought that each result reflected the various aspects of lupus nephritis. Of note, there was no difference of BUN and creatinine between IMQ-treated WT and *Padi4* KO mice, even though there was a significant difference in the proteinuria. One possible explanation is that PAD4-dependent neutrophil infiltration could be principally associated with the pathogenesis of proteinuria, not the reduction of renal functions. In human lupus nephritis, the poor outcome of renal sufficiency is associated with tubular intestinal damage (60). Since neutrophil infiltration was mainly found in glomerular lesions in IMQ-induced mice, we supposed that PAD4 would principally contribute to the glomerular lesions and proteinuria in this model. Further studies are required to explore the function of PAD4 in human lupus nephritis. In addition, the role of PAD4 in neutrophils and renal proximal tubular cells has been reported in acute kidney injury (61). Therefore, future studies with conditional KO mice specific for either neutrophils or tubular cells are also needed to dissect the cell-specific effect of PAD4 in lupus nephritis.

The importance of *PADI4* in the pathogenesis of SLE remains unclear. A genome wide association study (GWAS) revealed a number of critical genes and pathways for SLE (1). Although *PADI4* has not been identified as a GWAS risk gene for SLE, *PADI4* gene polymorphisms were reported to be associated with lupus nephritis (62). This may be due to the SLE case population with nephritis constitutes only a proportion of patients. With regard to immune-related nephritis, the fact that *PADI4* has been identified as a GWAS risk gene for IgA nephropathy supports the possibility that *PADI4* plays a critical role in immune complex-mediated nephritis (63). Recently, genetic variations in the *TNFAIP3* de-ubiquitinase domain, which is known to be associated with an increased risk of SLE, upregulates *PADI4* expression (64). These genetic studies supported the importance of *PADI4* in the pathogenesis of human lupus nephritis, and further human studies are required for unveiling the precise roles of *PADI4* in human diseases.

In summary, PAD4 promoted neutrophil infiltration into the kidneys and the development of nephritis in TLR7 agonist-induced lupus model mice. PAD4 was implicated in regulating the p38 MAPK pathway, which affects neutrophil adhesion to the kidneys. Our study demonstrated the importance of neutrophils in the pathogenesis of lupus nephritis, and the suppression of neutrophil adhesion and inhibition of the PAD4-p38 MAPK pathway may be a unique and promising therapeutic strategy against lupus nephritis.

## Data Availability Statement

The datasets generated for this study can be found in the GEO, GSE145422, https://www.ncbi.nlm.nih.gov/geo/query/acc.cgi?acc=GSE145422.

## Ethics Statement

The animal study was reviewed and approved by the ethics committee of The University of Tokyo Institutional Animal Care and Use Committee (15-P-069) and (P18-098).

## Author Contributions

HS, TK, TO, KYa, and KF designed experiments. NH conducted the experiments and acquired data. NH, HH, YN, and AS analyzed the data. AS, IG, and KYo provided animals. NH, HS, and KF wrote the manuscript. NH, HH, and YN prepared figures. All authors contributed to the article and approved the submitted version.

## Conflict of Interest

NH has received grants from GlaxoSmithKline. HS has received speaking fees from Takeda, BMS, Asahi Kasei, Sanofi, Eli Lilly, Janssen, Astellas, Pfizer, Chugai, UCB, and Daiichi-Sankyo. YN has received financial support or fees from BMS, Kissei, Mitsubishi Tanabe, and Pfizer. TK has received grants from GlaxoSmithKline. TO has received grants from Chugai. KYa has received fees for lecture from AbbVie, Ayumi, BMS, Chugai, Eisai, Janssen, Mitsubishi Tanabe, Ono, and UCB. KF has received grants, consulting fees, speaking fees, and/or honoraria from Mitsubishi Tanabe, BMS, Eli Lilly, Chugai, Janssen, Pfizer, Ono, AbbVie, Ayumi, Astellas, Sanofi, Novartis, Daiichi-Sankyo, Eisai, Asahi Kasei, MSD, and Japan Blood Products Organization. The remaining authors declare that the research was conducted in the absence of any commercial or financial relationships that could be construed as a potential conflict of interest.
