# Supplementary figures and images for: Peptidylarginine Deiminase 4 Promotes the Renal Infiltration of Neutrophils and Exacerbates the TLR7 Agonist-Induced Lupus Mice
Source: Front Immunol. 2020 Jun 23;11:1095. doi: 10.3389/fimmu.2020.01095 (PMC7324481; doi:10.3389/fimmu.2020.01095)

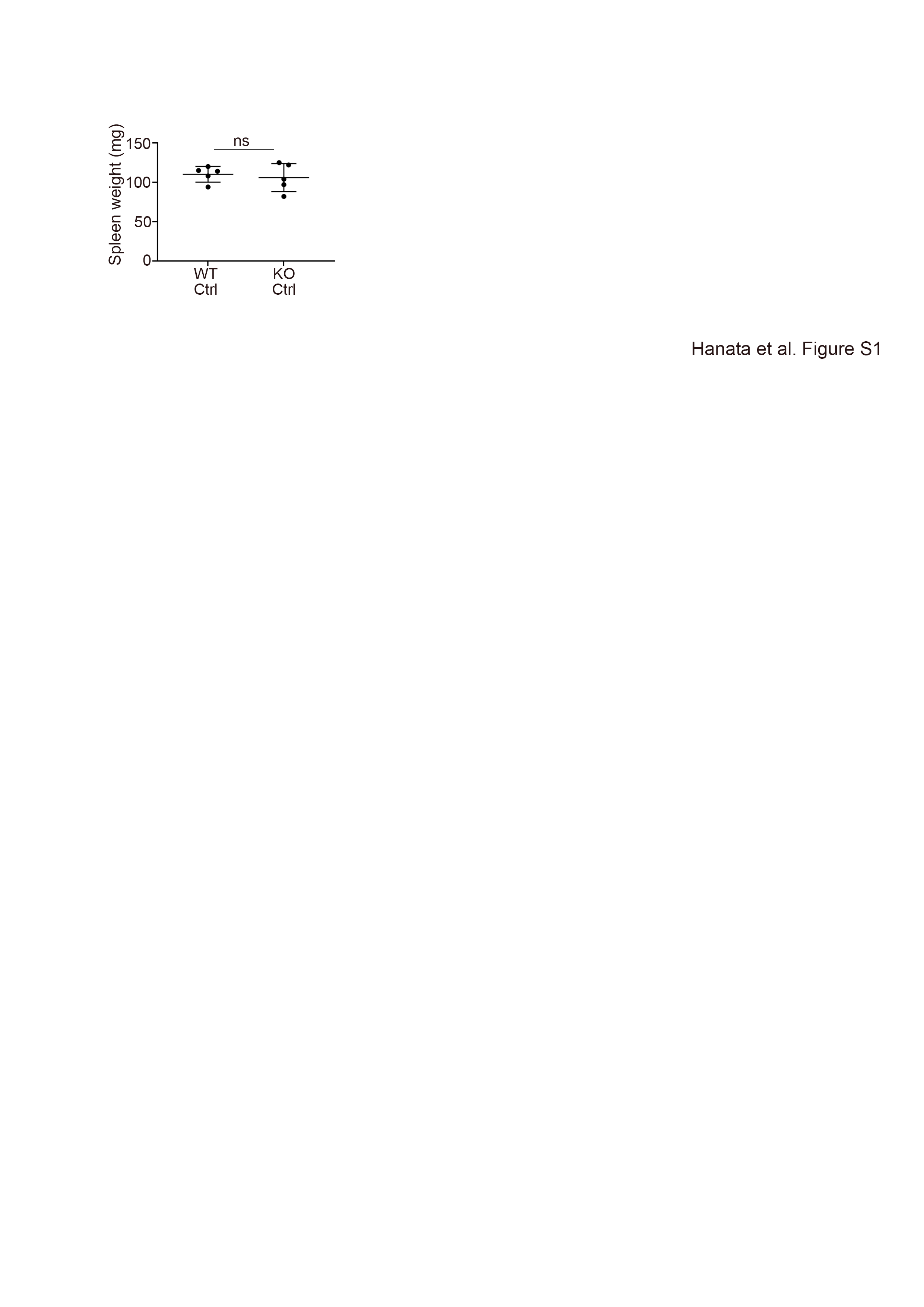

Supplement: Supplementary file 1 [file Image_1.tif]

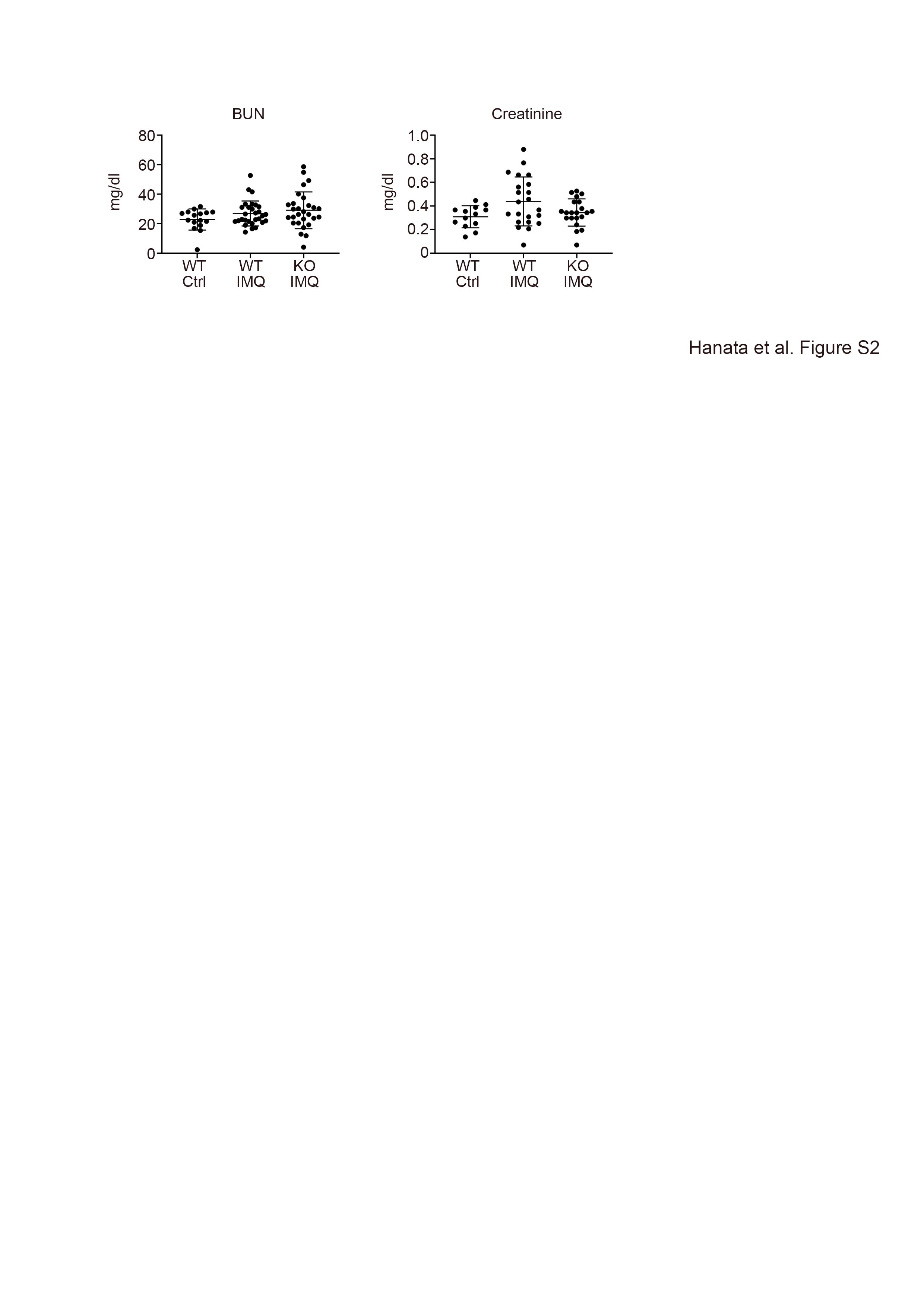

Supplement: Supplementary file 2 [file Image_2.tif]

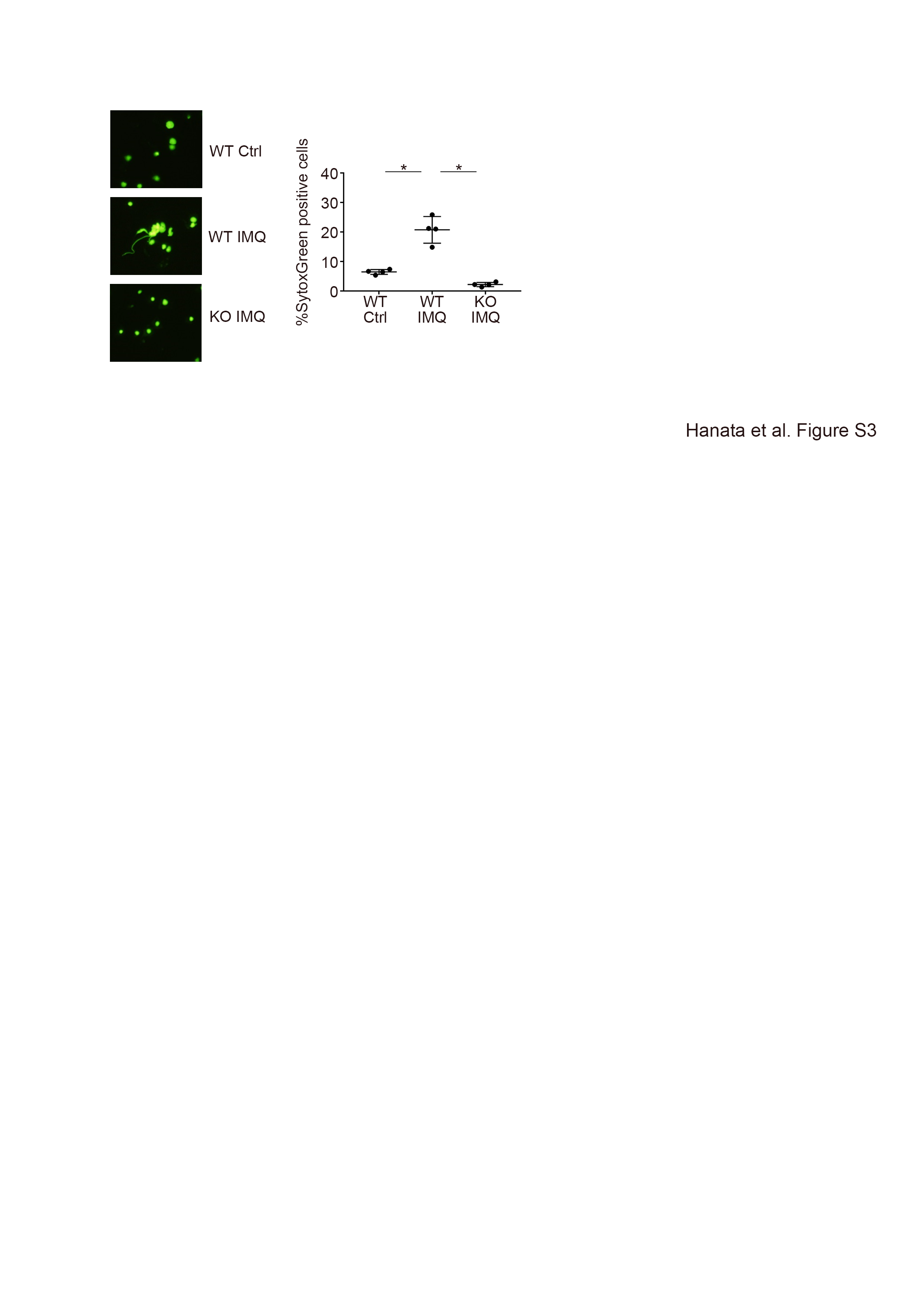

Supplement: Supplementary file 3 [file Image_3.tif]

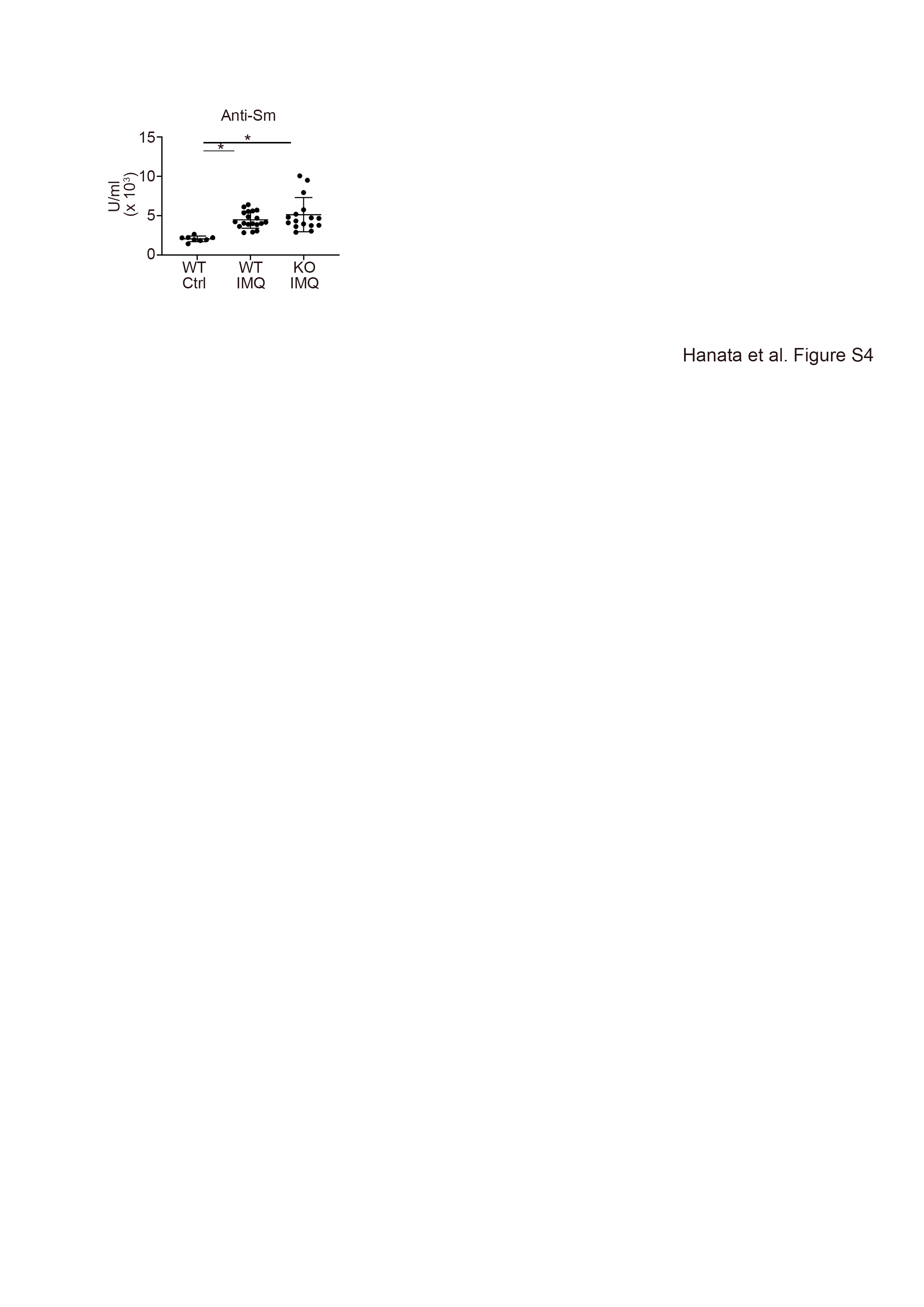

Supplement: Supplementary file 4 [file Image_4.tif]

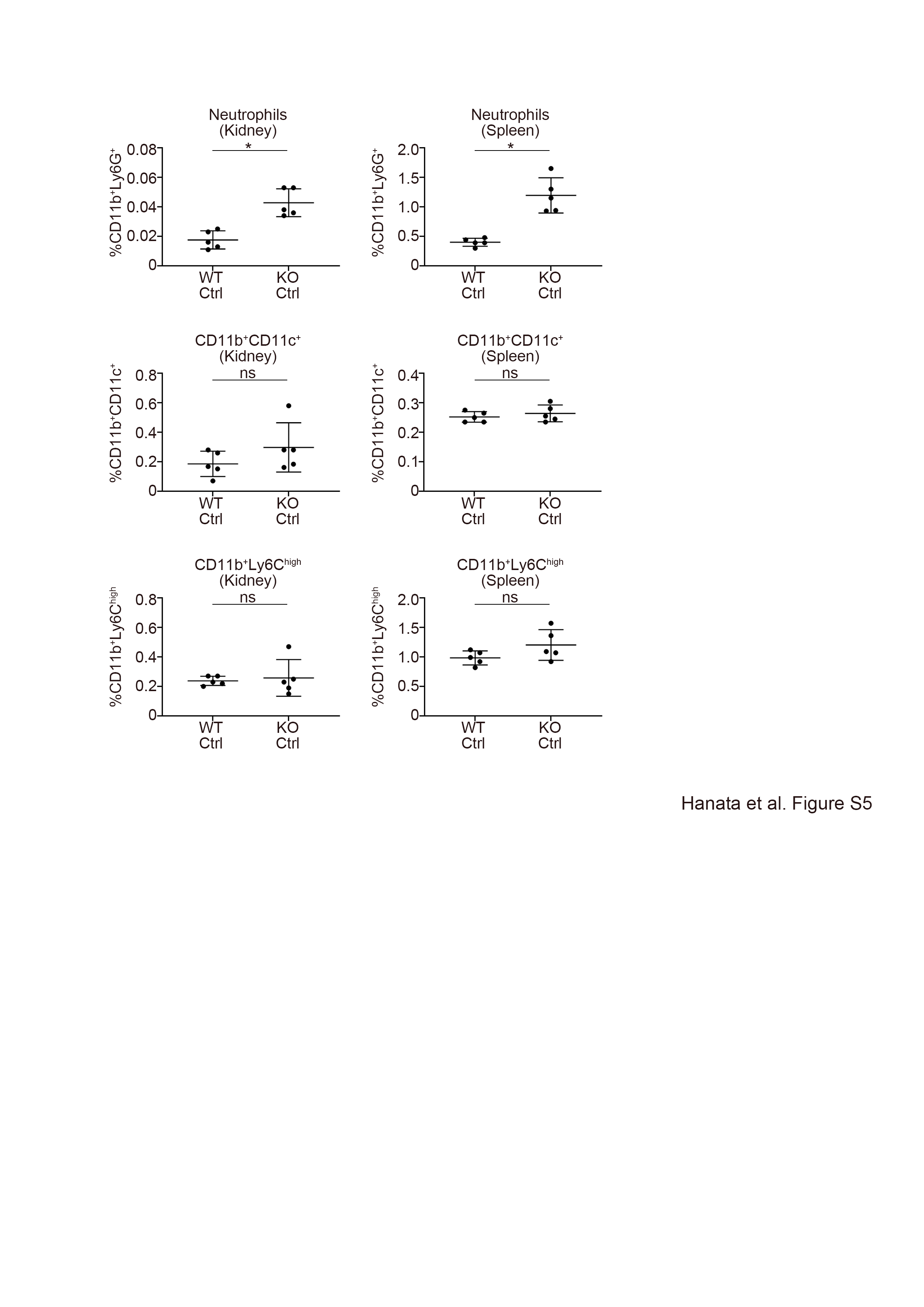

Supplement: Supplementary file 5 [file Image_5.tif]

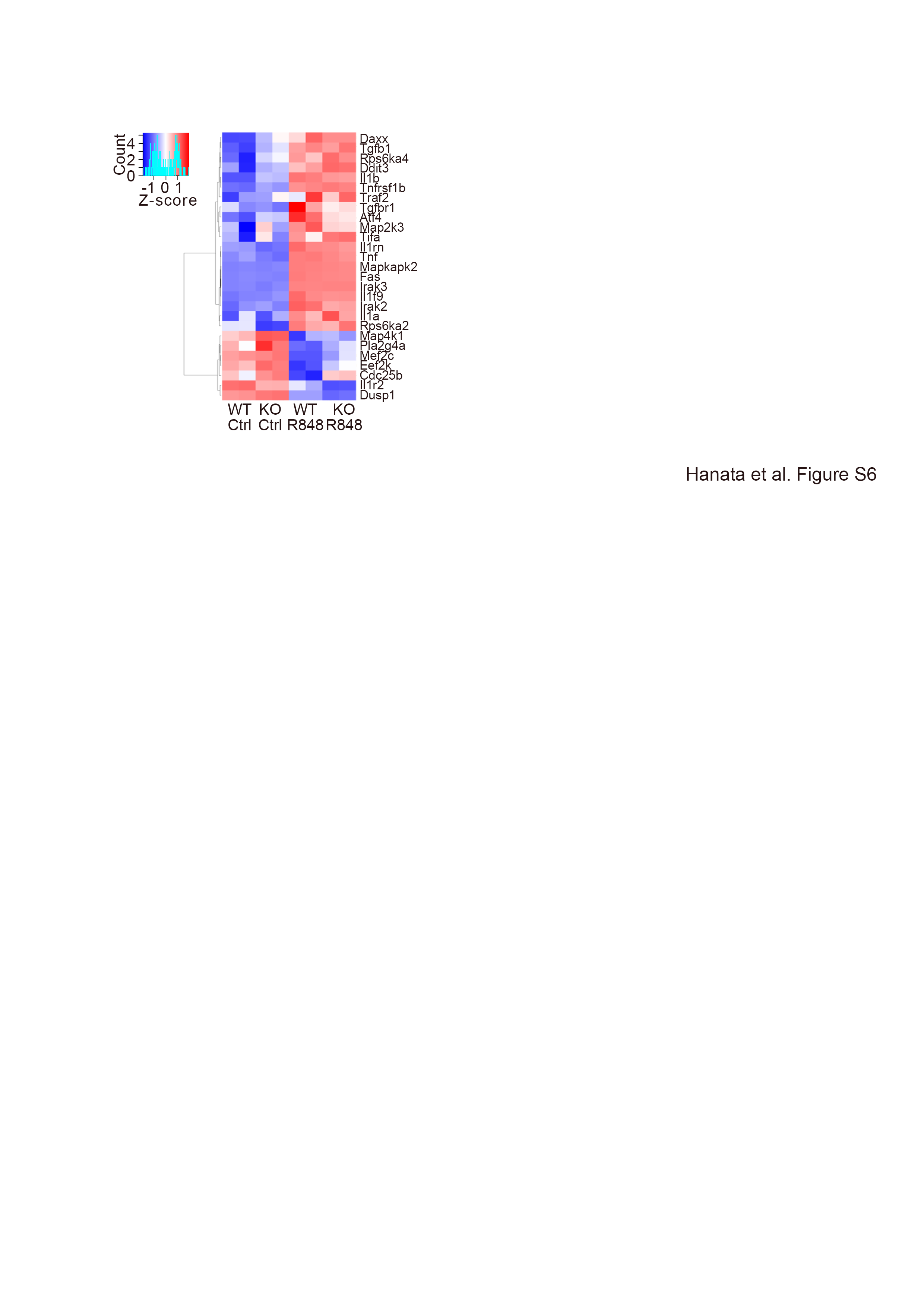

Supplement: Supplementary file 6 [file Image_6.tif]

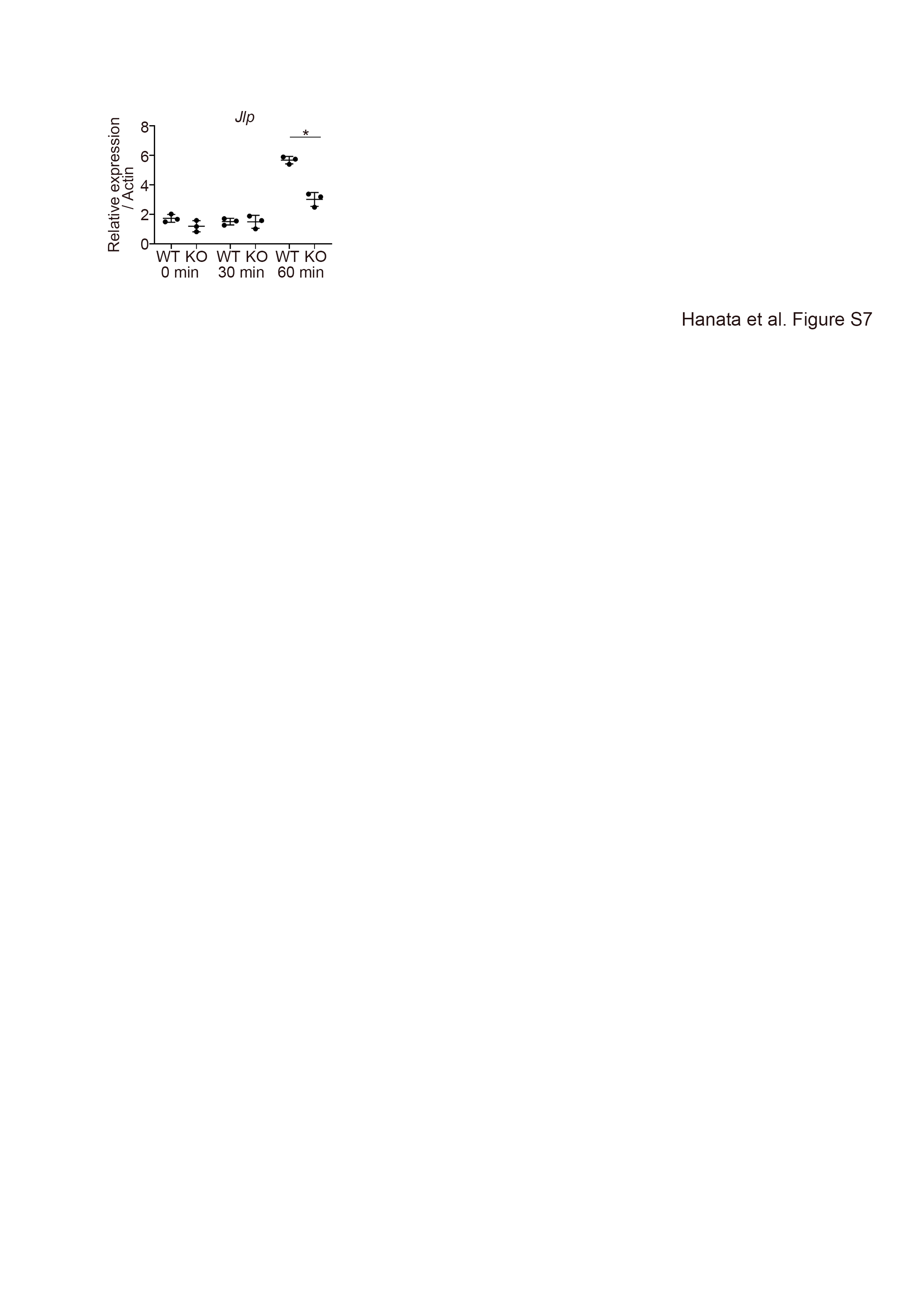

Supplement: Supplementary file 7 [file Image_7.tif]

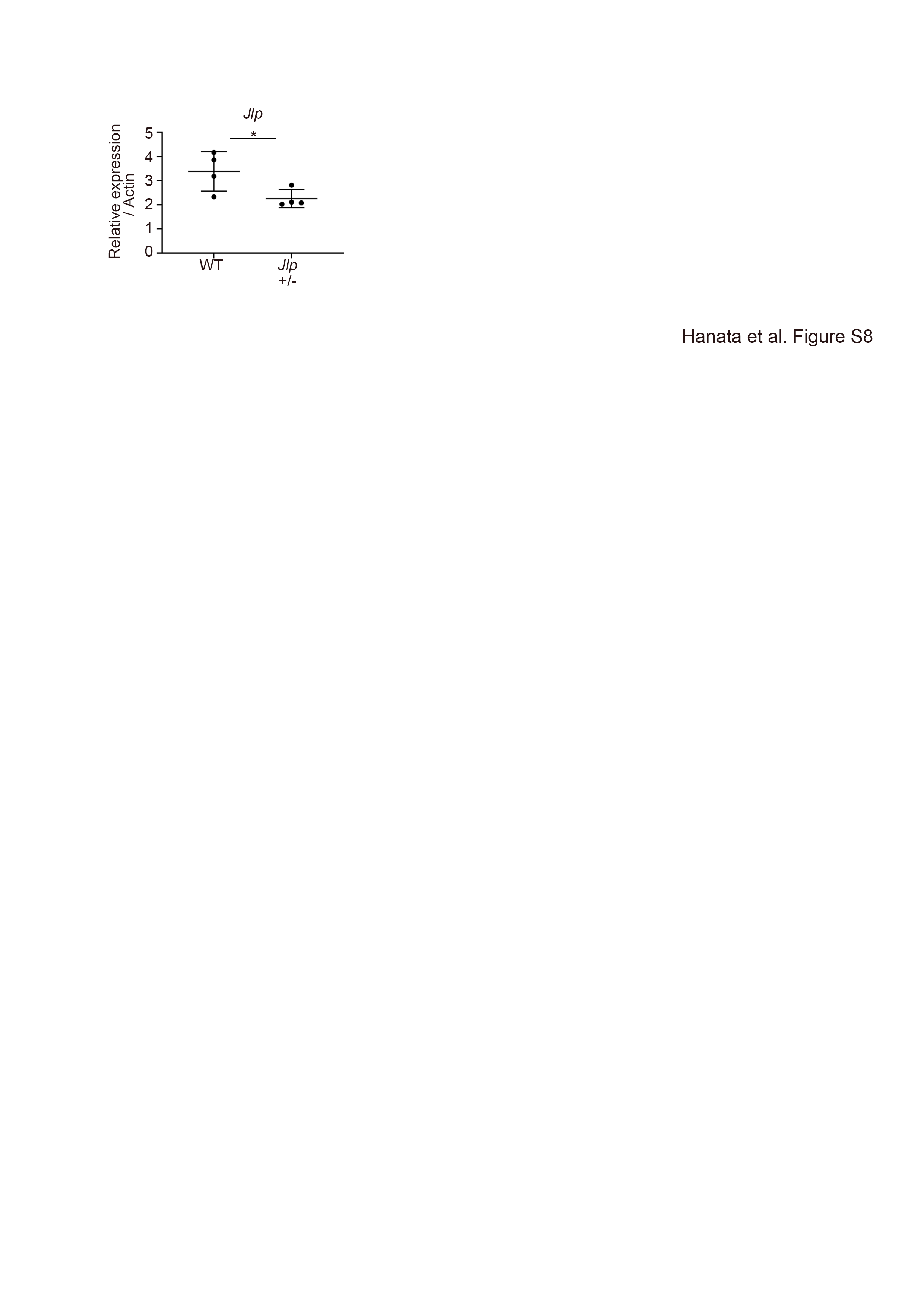

Supplement: Supplementary file 8 [file Image_8.tif]
