# Supplementary material for: Peptidylarginine Deiminase 4 Promotes the Renal Infiltration of Neutrophils and Exacerbates the TLR7 Agonist-Induced Lupus Mice
Source: Front Immunol. 2020 Jun 23;11:1095. doi: 10.3389/fimmu.2020.01095 (PMC7324481; doi:10.3389/fimmu.2020.01095)
Supplement: Supplementary file 9 [file Table_1.DOCX]

**Supplementary figure legends**

**Figure S1. Spleen weight of untreated mice**

Spleen weight of the untreated WT and *Padi4* KO mice was measured (n = 5 for WT and n = 5 for *Padi4* KO mice). Two-tailed unpaired t-test. ns, nonsignificant. All error bars represent ±SD.

**Figure S2. Serum BUN and Creatinine**

Left : Serum BUN of the WT control (WT Ctrl), IMQ-treated WT (WT IMQ), and IMQ-treated *Padi4* KO (KO IMQ) mice 8 weeks after IMQ treatment (WT Ctrl, n = 16; WT IMQ, n = 29; KO IMQ, n = 28). One-way ANOVA with Tukey’s multiple comparisons test.

Right : Serum Creatinine of the WT Ctrl, WT IMQ, and KO IMQ mice 8 weeks after IMQ treatment (WT Ctrl, n = 13; WT IMQ, n = 22; KO IMQ, n = 21). One-way ANOVA with Tukey’s multiple comparisons test. All error bars represent ±SD.

**Figure S3. NETosis *in vivo* in IMQ-treated mice**

Representative image of the bone marrow-derived neutrophils in WT control (WT Ctrl), IMQ-treated WT (WT IMQ), and IMQ-treated *Padi4* KO (KO IMQ) mice 8 weeks after IMQ treatment, which were incubated without any stimulation overnight, are shown (Left). DNA was stained with SytoxGreen. SytoxGreen positive cells were calculated using fluorescence microscopy (Right). (n = 4 for each groups). *p<0.05 (one-way ANOVA with Tukey’s multiple comparisons test). All error bars represent ±SD.

**Figure S4. Serum titers of anti-Sm antibody**

Serum titers of anti-Sm antibody in the serum of WT Ctrl, WT IMQ, and KO IMQ mice for 8 weeks are indicated (WT Ctrl, n = 8; WT IMQ, n = 25; KO IMQ, n = 16). *p < 0.05 (one-way ANOVA with Tukey’s multiple comparisons test). All error bars represent ±SD.

**Figure S5. Myeloid cells in the kidney and the spleen of the untreated mice**

The frequencies of CD11b^+^Ly6G^+^ cells (neutrophils), CD11b^+^CD11c^+^ cells, and CD11b^+^Ly6C^high^ cells (monocytes) in the kidneys and spleen cells of untreated mice (WT Ctrl, n = 5; KO Ctrl, n = 5). Data were analyzed by flow cytometry. *p < 0.05 (two-tailed unpaired t-test). All error bars represent ±SD.

**Figure S6. Heatmap for read counts of genes related to the p38 MAPK pathway in IPA with hierarchical clustering**

Neutrophils (1 × 10^6^/ml) were incubated in a 24-well plate and left unstimulated or stimulated with or without 1 µg/ml of R848 for 6 hours. A heatmap for read counts of genes related to the p38 MAPK pathway in IPA with hierarchical clustering is shown. Read counts were normalized as count per million and scaled by row Z-score.

**Figure S7. *Jlp* mRNA in WT and *Padi4* KO neutrophils following LPS stimulation**

Real-time PCR analysis of the expression of *Jlp* mRNA in WT and *Padi4* KO neutrophils at 0, 30, and 60 minutes after LPS (1 µg/ml) stimulation (n = 3 per group). Results were normalized to *Actb*. *p < 0.05 (two-tailed unpaired t-test)

**Figure S8. mRNA expression of *Jlp* in *Jlp* +/- neutrophils**

Real-time PCR analysis of *Jlp* mRNA expression in bone marrow-derived WT and *Jlp* +/- neutrophils (n=4 per group). Results were normalized to *Actb*. *p<0.05 (two-tailed unpaired t-test).
